# Supplementary material for: Pollinator Proboscis Length Plays a Key Role in Floral Integration of Honeysuckle Flowers (Lonicera spp.)
Source: Plants (Basel). 2023 Apr 12;12(8):1629. doi: 10.3390/plants12081629 (PMC10144162; doi:10.3390/plants12081629)
Supplement: Supplementary file 1 [file plants-12-01629-s001.zip › Table S1.pdf]

Table S1 Information on the study sites, pollinator information (pollinator species and guilds, pollinator proboscis length and visitation rate of each pollinator guild) and population sizes for the 11 studied *Lonicera* species in each natural population.

Species	GPS coordinate/ site abbreviation	Altitude (m)	Pollinator species	Pollinator guild	Proboscis length for each pollinator (mm)	Visitation rate (visiting number/flower/20min, N=30)	Population sizes
L. chrysantha	33°59′1″(N) 107°16′5″(E) TB-SX	2106	*Apis cerana*	Honeybee	3.045±0.095, N=28	0.045±0.0085	> 200
			Bombus pyrosoma, B. flavus	Bumblebee	7.817±0.201, N=23	0.057±0.0065	
L.elisae	34°00′2″(N) 107°17′5″(E) TB-SX	1829	*Apis cerana*	Honeybee	3.045±0.095, N=28	0.0033±0.0019	> 100
			Osmia Sp.	Leafcutter bee	8.864±0.247, N=19	0.0824±0.0103	
L. ferdinandi	34°04′2″(N) 107°21′2″(E) TB-SX	1622	*Apis cerana*	Honeybee	3.045±0.095, N=28	0.0486±0.0067	> 100
			Bombus picipes	Bumblebee	7.817±0.201, N=23	0.01±0.0042	

L. gynochlamydea	31°25′5″(N) 110°21′5″(E) SNJ-HB	1456	*Lasioglossum calceatum, L. scitulum*	Solitary bee	2.449±0.104, N=20	0.012±0.0043	> 100
			Apis cerana	Honeybee	3.045±0.095, N=28	0.0574±0.0024	
			Bombus festivus, B. lucorum	Bumblebee	7.817±0.201, N=23	0.032±0.0079	
			Ceratina chinensis	Solitary bee	2.449±0.104, N=20	0.0132±0.0019	
L. japonica	33°49′3″(N) 107°30′1″(E) TB-SX	1372	*Xylocopa sinensis, Bombus trifasciatus, Theretra nessus*	Bumblebee	7.817±0.201, N=23	0.0567±0.0075	> 100
			Macroglossum pyrrhosticta, M. bombylans, M. bombylans, Theretra nessus	Hawkmoth	41.15±1.044, N=6	0.011±0.0043	

L. maackii	34°04′2″(N) 107°21′2″(E) TB-SX	1622	*Apis cerana*	Honeybee	3.045±0.095, N=28	0.103±0.0095	> 300
L. pileata	31°26′5″(N) 110°23′2″(E) SNJ-HB	1178	*Apis cerana*	Honeybee	3.045±0.095, N=28	0.0256±0.0131	> 100
			Bombus festivus, B. pyrosoma	Bumblebee	7.817±0.201, N=23	0.0556±0.0212	
			Ceratina chinensis	Solitary bee	2.449±0.104, N=20	0.0254±0.0063	
L. standishii	34°04′2″(N) 107°21′2″(E) TB-SX	1647	*Apis cerana*	Honeybee	3.045±0.095, N=28	0.0813±0.0101	> 200
			Bombus lucorum, B. picipes, Episyrphus balteata	Bumblebee	7.817±0.201, N=23	0.005±0.0029	
L.tangutica	33°59′17″(N) 107°16′52″(E) TB-SX	2106	*Bombus picipes*	Bumblebee	7.817±0.201, N=23	0.105±0.0244	> 100

L. tragophylla	34°01′2″(N) 107°18′3″(E) TB-SX	1648	*Theretra nessus, Macroglossum bombylans, M. pyrrhosticta*	Hawkmoth	41.15±1.044, N=6	0.00959±0.0029	> 100
L. webbiana	34°00′4″(N) 107°17′5″(E) TB-SX	1844	*Apis cerana*	Honeybee	3.045±0.095, N=28	0.0762±0.0074	> 100
			Episyrphus balteata	Bumblebee	7.817±0.201, N=23	0.00815±0.0042	
			Lasioglossum calceatum	Solitary bee	2.449±0.104, N=20	0.019±0.0045	
