# Supplementary material for: Pollinator Proboscis Length Plays a Key Role in Floral Integration of Honeysuckle Flowers (Lonicera spp.)
Source: Plants (Basel). 2023 Apr 12;12(8):1629. doi: 10.3390/plants12081629 (PMC10144162; doi:10.3390/plants12081629)
Supplement: Supplementary file 1 [file plants-12-01629-s001.zip › Table S2.pdf]

Table S2 Comparison of evolutionary models in PGLS on association of floral integration (INT) with floral traits (corolla tube length, stigma height, upper lip length and lower lip length) and composite pollinator proboscis length (PLa). These models were modelled using Brownian motion (BM), Pagel's lambda (PL), as well as Ornstein-Uhlenbeck (OU). Models were evaluated by Akaike information criterion (AIC). Best-fitting models are shown in bold. "-" means model was not significant ($P > 0.05$).

Model		AIC of each phylogenetic model		
predictor variable	response variable	BM	PL	OU
corolla tube length	INT	-	NA	**76.94**
stigma height	INT	-	**77.35**	77.94
upper lip length	INT	-	**72.74**	75.88
lower lip length	INT	-	**73.72**	76.85
proboscis length	INT	-	**73.54**	75.42
