# Supplementary material for: Pollinator Proboscis Length Plays a Key Role in Floral Integration of Honeysuckle Flowers (Lonicera spp.)
Source: Plants (Basel). 2023 Apr 12;12(8):1629. doi: 10.3390/plants12081629 (PMC10144162; doi:10.3390/plants12081629)
Supplement: Supplementary file 1 [file plants-12-01629-s001.zip › Table S3.pdf]

Table S3 Statistical results of the comparison of 14 phylogenetic structural equation models (PSEMs) based on AIC to determine the pathway that pollinators proboscis length mediate floral integration for the 11 *Lonicera* flowers. The models were constructed based on every possible combination of pathways among modules; we supposed that pollinator proboscis length was related to a single, two and three of the functional modules (accessibility, efficiency and attractiveness), respectively. The relationship between upper lip length and lower lip length was defined as being correlated errors, thus we only show the direct paths in each model. The best model was selected as the one with the lowest AIC value among those with a *P* value > 0.05 derived from Fisher's C test statistic and tests of d-separation and with no statistically significant missing paths between variables (indicated in the "Missing paths" column). The best-fitting SEM is marked in bold.

No.	Condition	Direct paths			Fisher's C	*P*-value	AIC	Missing paths*
		1st	2nd	3rd				
1	single	CTL*	SH	ULL	59.301	31.301	0	-
2	single	CTL*	SH	DLL	53.511	25.511	0	-
**3**	**single**	**SH***	**CTL**	**ULL**	**28.338**	**0.338**	**0.844**	**None**
4	single	SH*	CTL	DLL	37.245	9.245	0.01	None
5	single	ULL*	CTL	SH	214.246	186.246	0	-
6	single	DLL*	CTL	SH	214.246	186.246	0	-
7	two	CTL*	SH*	ULL^{1st}	267.048	241.048	0	-
8	two	CTL*	SH*	DLL^{1st}	261.258	235.258	0	-

9	two	CTL*	SH*	ULL^{2nd}	248.727	222.727	0	-
10	two	CTL*	SH*	DLL^{2nd}	248.727	222.727	0	-
11	two	CTL*	ULL*	SH^{1st}	95.324	69.324	0	-
12	two	CTL*	DLL*	SH^{1st}	117.736	91.736	0	-
13	two	CTL*	ULL*	SH^{2nd}	242.694	216.694	0	-
14	two	CTL*	DLL*	SH^{2nd}	246.976	220.976	0	-
15	two	SH*	ULL*	CTL^{1st}	87.309	61.309	0	-
16	two	SH*	DLL*	CTL^{1st}	109.17	83.17	0	-
17	two	SH*	ULL*	CTL^{2nd}	288.136	262.136	0	-
18	two	SH*	DLL*	CTL^{2nd}	269.117	243.117	0	-
19	three	CTL*	SH*	ULL*	339.053	315.053	0	-
20	three	CTL*	SH*	DLL*	387.508	363.508	0	-

"*" means the floral trait was direct correlated with pollinator proboscis. In each path, ordinal number demonstrates which the floral trait was connected to the trait in the third column. "-" indicates "not applicable". Floral traits in models were corolla tube length (CTL), anther height stigma height (SH), upper and lower corolla lip length (ULL and LLL).
