# Supplementary figures and images for: Pollinator Proboscis Length Plays a Key Role in Floral Integration of Honeysuckle Flowers (Lonicera spp.)
Source: Plants (Basel). 2023 Apr 12;12(8):1629. doi: 10.3390/plants12081629 (PMC10144162; doi:10.3390/plants12081629)

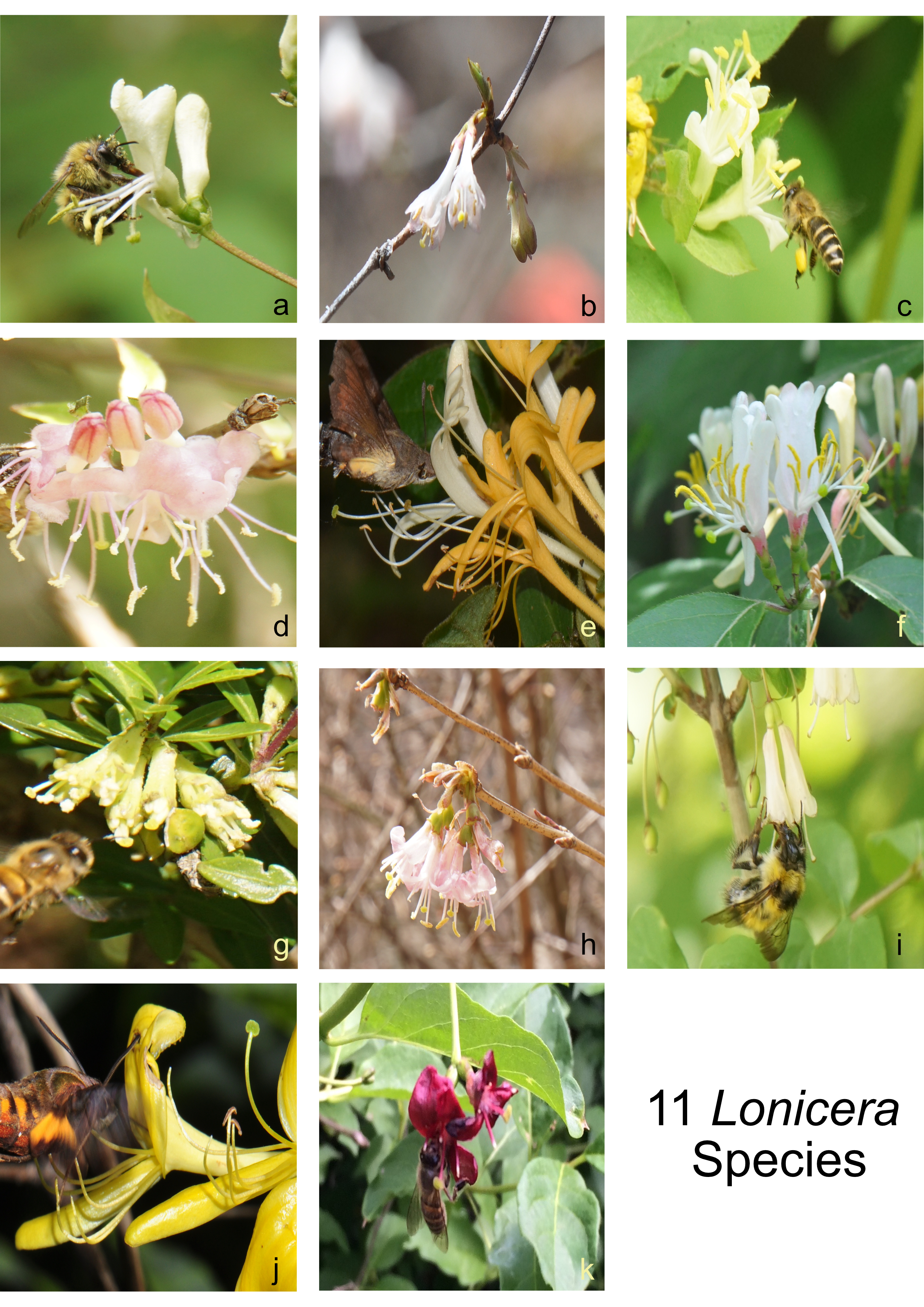

Supplement: Supplementary file 1 [file plants-12-01629-s001.zip › Figure S1.jpeg]
